# A theoretical analysis of the vibrational modes of ammonium metavanadate†

**DOI:** 10.1039/d3ra02053c

**Published:** 2023-05-26

**Authors:** Qing Guo, Xiao-Yan Liu, Si-Cheng Liu, Yi-Ning Li, Yi Yin, Peng Zhang

**Affiliations:** a School of Space Science and Physics, Shandong University Weihai 264209 China zhangpeng@sdu.edu.cn

## Abstract

Vanadium(v) is an extremely rare and precious metal, mainly used in aerospace equipment and new energy construction. However, an efficient, simple, and environmentally friendly method for separating V from its compounds is still lacking. In this study, we used first-principles density functional theory to analyse the vibrational phonon density of states of ammonium metavanadate and simulated its infrared absorption and Raman scattering spectra. By analysing the normal modes, we found that the V-related vibration has a strong infrared absorption peak at 711 cm^−1^, while other significant peaks above 2800 cm^−1^ are from N–H stretching vibrations. Therefore, we propose that providing high-power terahertz laser radiation at 711 cm^−1^ may facilitate the separation of V from its compounds through phonon–photon resonance absorption. With the continuous progress of terahertz laser technology, this technique is expected to be developed in the future, and it may offer new technological possibilities.

## Abstract 2

The peak at 711 cm^−1^ is from the V–O stretching vibrational mode. It is selected for ideal photon–phonon resonance absorption frequency.

## Graphical abstract

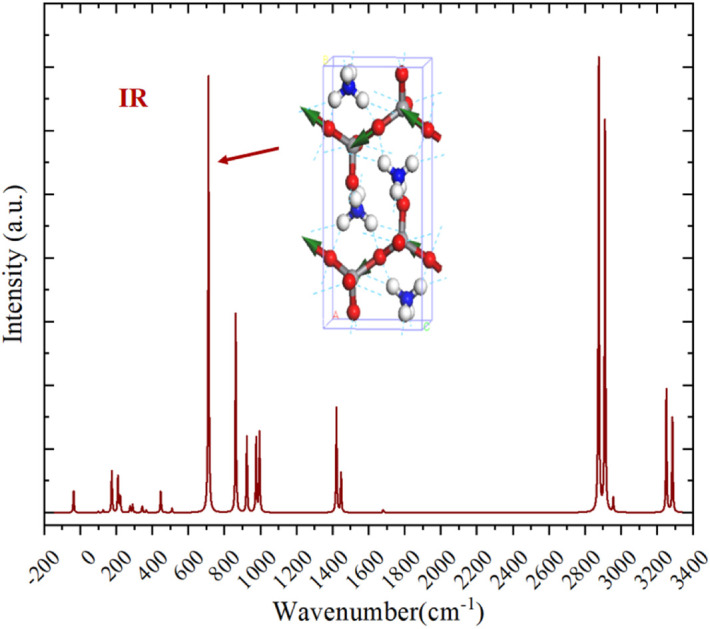

## Introduction

Vanadium(v) is an extremely rare metal with high melting and boiling points. It is resistant to both hydrochloric acid and sulfuric acid and outperforms most stainless steels in some ways. Because of its extensive utility, it is known as the metal ‘vitamin’. V is mainly used in the production of high-strength low-alloy steels, specialised steels, and aerospace alloys, as well as in other applications.^1–3^ In the field of aerospace, the excellent improvement effect of V in titanium alloys has been discovered, making it useful in body structures and jet engines.^4–6^ The global market for V redox flow batteries is also expected to grow at an annual rate of 59.7% between 2018 and 2025.^7^ Considering the production and demand data of V for the energy technology industry specifically, the demand for V is expected to increase by up to 73% by 2050.^8^ This dramatic increase will be due to the growing demand for new materials, making mining and recycling of renewable resources strategically important for many countries.

Currently, there are various methods of extracting V directly from ores and secondary raw materials, including sodium roasting–water leaching V extraction^9–13^ and calcification roasting–acid leaching V extraction.^14–18^ However, these methods have disadvantages, such as causing pollution due to the use of various additives in the roasting and leaching processes and high costs. It is worth noting that China is one of the countries with the richest V mineral resources.^19^ However, an efficient and environmentally friendly method of extracting V has not yet been developed,^20^ and there is a lack of research on the recovery of secondary V resources, such as titanium–ferromagnetic slag.

Since the discovery of the crystal structure of ammonium metavanadate (NH_4_VO_3_) in 1950,^21^ many studies using infrared (IR) and Raman spectroscopy have focused on V in NH_4_VO_3_.^22–30^ Among these studies, Waal and Twu *et al.* investigated the pyrolysis process of NH_4_VO_3_ under the action of N_2_ and NH_3_ + H_2_O using Raman spectroscopy at the molecular level.^24,27^ However, there has been a lack of theoretical study of the lattice dynamic processes based on vibrational spectroscopy. In this work, we simulated the vibrational spectrum of NH_4_VO_3_ and analysed the normal modes. Through our analysis, we were able to assign V-related vibrational peaks and determine the IR-active modes of V in the NH_4_VO_3_ spectrum. Based on these findings, we propose a new method to assist V separation using photon–phonon resonance absorption (PPRA).

### Simulation methods

We performed geometry optimisation and phonon calculations using the CASTEP code,^31^ which implements the first-principles density functional theory method. We adopted the generalised gradient approximation in the form of the revised Perdew–Burke–Ernzerhof (RPBE) exchange–correlation functional because the gradient of electron density in NH_4_VO_3_ varies widely.^32^ The convergence tolerance for energy and self-consistent field (SCF)was set to 1 × 10^−9^ eV per atom to eliminate virtual frequencies. The energy cut-off was set to 750 eV and the *K*-point mesh to 3 × 1 × 2 to calculate phonons using a norm-conserving pseudopotential and the linear response method. The property of polarisability, IR and Raman spectra were calculated. The simulated spectra can be compared with experimental data and the vibrational normal modes from the phonon calculation can be used for assignments.

NH_4_VO_3_ has a pyroxene (Si_2_O_6_) structure, and its primitive cell contains 36 atoms with space group *Pbcm*.^21,33,36^ The crystal consists of four NH_4_^+^ clusters and two VO_3_^−^ ribbons oriented in one direction. N_3_^−^ is bonded in tetrahedral geometry to four H_1_^+^ atoms in NH_4_ clusters, while V_5_^+^ is bonded to four O_2_^−^ atoms to form corner-sharing VO_4_ tetrahedra in each VO_3_ ribbon. In the analysis of vibrational modes, intermolecular vibrations were classified as translation and rotation modes, while intramolecular vibrations were classified as bending and stretching modes. The study focused on the V-related vibrational modes for further analysis and assignments.

## Results and discussion

**Fig. 1:**
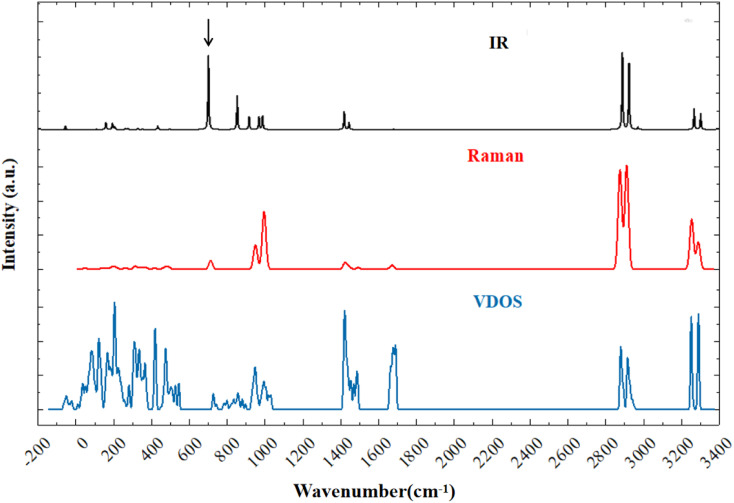
Simulated vibrational spectra of ammonium metavanadate: IR spectrum (black), Raman spectrum (red), and VDOS (blue).

Fig. 1 shows the simulated infrared (IR) spectrum, Raman spectrum, and phonon vibrational density of states (VDOS) of NH_4_VO_3_. Considering the normal vibrations at the gamma point, there are 108 normal modes for a 36-atom primitive cell, corresponding to 36 × 3 = 108 dispersion curves that make up the VDOS. Excluding three acoustic branches, there are 105 optical branches that can be detected by IR absorption or Raman scattering through phonon–photon coupling.

**Fig. 2:**
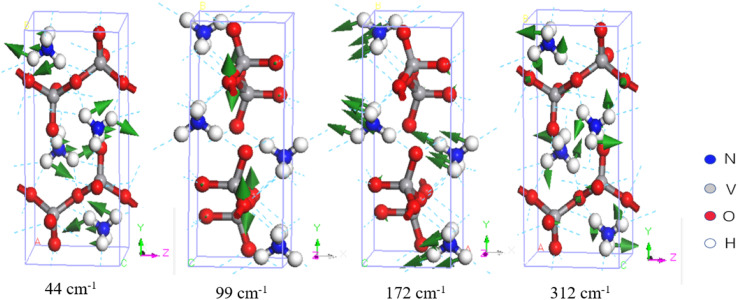
Four examples of vibrational modes at 44–312 cm^−1^. The green arrows represent the direction of vibration, where the size of each arrow is proportional to the vibrational amplitude. The number below each mode indicates its wavenumber.

Due to the unique structure of NH_4_VO_3_, its IR- and Raman-active phonons are fully complementary. Table 1 presents a comparison between the calculated normal modes and the experimental IR-active and Raman-active vibrational modes. Park *et al.* reported the Raman spectrum of NH_4_VO_3_ in 1989 and assigned the peaks at 85.5, 149, 170, 195, and 209 cm^−1^ to translational modes, and the peaks at 269 and 311 cm^−1^ to rotational modes.^36^ Similarly, Du *et al.* reported the Raman and IR spectra and assigned the Raman-active peaks at 260 and 210 cm^−1^ to V–O bending vibrations,^30^ while Onodera *et al.* assigned the peaks at 205 and 255 cm^−1^ (Raman) and the peak at 223 cm^−1^ (IR) to V–O–V bending vibrations.^34^ In this work, we identified corresponding Raman-active vibrational modes at 44, 84, 137, 152, 195, 215, 261, and 312 cm^−1^, while the IR-active vibrational modes were at 99, 126, 172, 207, and 221 cm^−1^. In the low-frequency band, the vibrational modes are intermolecular collective vibrations. Our vibrational analysis confirmed that the modes at 44 and 84 cm^−1^ belong to NH_4_^+^ rotations, the modes at 99, 126, and 137 to VO_3_^−^ rotations, the modes at 152, 172, 195, and 207 to NH_4_^+^ translations, and the modes at 215, 221, 261, and 312 cm^−1^ to skeletal vibrations, where the two ionic groups vibrate together. Fig. 2 presents examples to illustrate these four types of vibrational modes.

**Table 1:** Comparison of calculated IR- or Raman-active normal modes with experimental data, with the relative assignments listed in the last column

Normal mode (cm^−1^)	Active	IR exp	Raman exp	Vibrational mode
44	Raman			NH_4_^+^ rotation
84	Raman		85.5^d^	NH_4_^+^ rotation
99	IR			VO_3_^−^ rotation
126	IR			VO_3_^−^ rotation
137	Raman		149^d^	VO_3_^−^ rotation
152	Raman		170^d^	NH_4_^+^ translation
172	IR			NH_4_^+^ translation
195	Raman		205^b^, 195^d^	NH_4_^+^ translation
207	IR			NH_4_^+^ translation
215	Raman		210^a^, 209^d^	Skeletal rotation
221	IR	223^b^		Skeletal rotation
261	Raman		260^a^, 255^b^, 269^d^	Skeletal rotation
312	Raman		311^d^	Skeletal rotation
317	Raman		315^b^, 326^d^	VO_3_^−^ bending
342	Raman		342^b^, 344^d^	VO_3_^−^ bending
367	Raman		385^a^, 380^b^	VO_3_^−^ bending
446	IR			Skeletal rotation
469	Raman			Skeletal rotation
489	Raman		496^a^, 497^b^, 497^d^	Skeletal rotation
711	IR	690^b^		VO_3_^−^ stretching
712	Raman		647^a^, 643^b^, 646^d^	VO_3_^−^ stretching
862	IR	895^a^, 850^b^		VO_3_^−^ stretching
898	Raman		898^a^, 895^b^, 897^d^	VO_3_^−^ stretching
924	IR	935^a^, 935^b^		VO_3_^−^ stretching
948	Raman		928^a^, 925^b^ 936^d^	VO_3_^−^ stretching
959	Raman			VO_3_^−^ stretching
975	IR			VO_3_^−^ stretching
994	IR			VO_3_^−^ stretching
994	Raman			VO_3_^−^ stretching
1418	Raman		1370^b^, 1384^d^	NH_4_^+^ bending
1420	IR	1416^a^, 1412^b^, 1406^c^		NH_4_^+^ bending
1420	IR	1414^c^		NH_4_^+^ bending
1423	Raman		1420^b^, 1435^d^	NH_4_^+^ bending
1441	Raman		1440^b^, 1444^d^	NH_4_^+^ bending
1446	IR	1422^c^		NH_4_^+^ bending
1490	Raman			NH_4_^+^ bending
1662	Raman			NH_4_^+^ bending
1673	Raman		1650^b^, 1654^d^	NH_4_^+^ bending
2874	IR	2839^c^		NH_4_^+^ stretching
2875	Raman			NH_4_^+^ stretching
2876	IR	2790^a^, 2800^b^, 2926^c^		NH_4_^+^ stretching
2910	Raman		2920^b^, 2920^d^	NH_4_^+^ stretching
2911	IR	2950^a^, 2980^b^, 3019^c^		NH_4_^+^ stretching
2912	Raman			NH_4_^+^ stretching
2956	IR	3122^c^		NH_4_^+^ stretching
3251	IR	3200^a^, 3190^b^, 3207^c^		NH_4_^+^ stretching
3253	Raman		3050^b^, 3050^d^	NH_4_^+^ stretching
3285	IR			NH_4_^+^ stretching
3285	Raman			NH_4_^+^ stretching
3289	Raman			NH_4_^+^ stretching

a Ref. 30.

b Ref. 34.

c Ref. 35.

d Ref. 36.

**Fig. 3:**
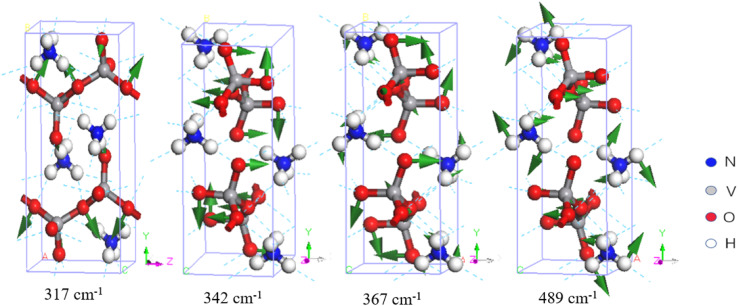
Four examples of vibrational modes at 317–489 cm^−1^. The first three modes represent VO_3_^−^ bending, and the last one represents a skeletal rotation.

In Fig. 3, three Raman-active normal modes are evident at 317, 342, and 367 cm^−1^, which are bending vibrations of the V–O bond. Onodera *et al.* compared NH_4_VO_3_ with KVO_3_ and assigned a peak at 375 cm^−1^ to the hydrogen bond vibration between NH_4_^+^ and VO_3_^−^.^34^ Du *et al.* attributed the mode at 385 cm^−1^ to the V=O bending vibration.^30^ Park *et al.* discovered two Raman peaks at 326 and 344 cm^−1^, one of medium intensity and the other a shoulder peak.^36^ According to Fig. 1, the Raman peaks in this region are very weak.

In the IR spectrum, Du *et al.* assigned various combined vibrations of V–O bonds between 500 and 850 cm^−1^ and a symmetric vibration of a Raman-active peak at 496 cm^−1^.^30^ Similarly, Onodera and Park assigned the Raman peak at 497 cm^−1^ to the symmetric vibration.^34,36^ They tentatively assigned the peaks using group theory. Table 1 shows that the simulated normal mode at 489 cm^−1^ corresponds to a skeletal rotation, as shown in Fig. 3.

**Fig. 4:**
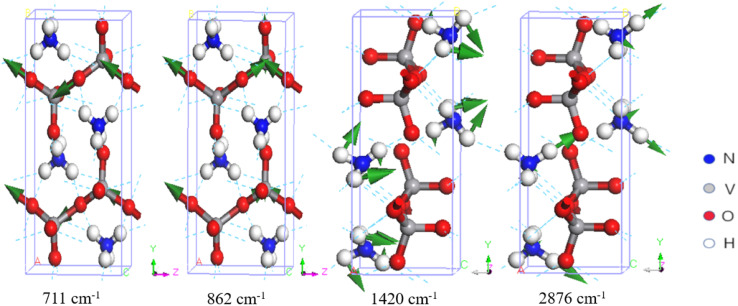
Four examples of vibrational modes. The first two correspond to VO_3_^−^ stretching, the third to NH_4_^+^ bending, and the last to NH_4_^+^ stretching.

There are 10 normal modes ranging from 700 to 1000 cm^−1^, which are all related to V–O stretching. Onodera and Park assigned three Raman peaks at 643, 895, and 925 cm^−1^, and three at 646, 897, and 936 cm^−1^, to asymmetric and symmetric V–O stretching vibrations, respectively. Onodera also observed IR peaks at 690, 850, and 935 cm^−1^. Du assigned two IR peaks at 895 and 935 cm^−1^ to V–O stretching. Our simulations are in good agreement with the experiments. We found five IR-active modes at 711, 862, 924, 975, and 994 cm^−1^, and five Raman-active modes at 712, 898, 948, 959, and 994 cm^−1^. Note that the IR-active mode at 994 cm^−1^ is not the same as the Raman-active mode at 994 cm^−1^. These two wavenumbers are not degenerate energies; they appear equal only due to number rounding. As shown in Table 1, these modes all correspond to V–O stretching. Two examples are shown in Fig. 4. The IR-active modes at 711 and 862 cm^−1^ correspond to the experimental IR peaks at 690, 850 cm^−1^ (ref. 34), and 895 cm^−1^ (ref. 30). The dynamic process of V–O asymmetric stretching at 711 cm^−1^ is presented in the ESI files.† Compared with the other V-related IR-active modes at 862, 924, 975, and 994 cm^−1^, the intensity ratios to 711 cm^−1^ are 45.55%, 17.23%, 17.02%, and 18.63%, respectively. It is clear that the IR peak at 711 cm^−1^ is very strong, which means that the PPRA effect at this peak should be very efficient. If a terahertz laser at this frequency is applied to NH_4_VO_3_, the V–O bonds will absorb the radiation energy efficiently, which may potentially break the chemical bonds and facilitate the separation of V from NH_4_VO_3_.

In the higher-frequency region ranging from 1418 to 3289 cm^−1^, we found that all vibrations are related to the NH_4_^+^ group due to the low mass of the ions. Waal *et al.* attributed 1414 and 1422 cm^−1^ to the triply degenerate bending vibrations of NH_4_^+^ and pointed out a shoulder peak at 1406 cm^−1^.^35^ Onodera *et al.* suggested that the Raman peaks at 1420, 1440, and 1650 cm^−1^ represent NH_4_^+^ bending, while the peak at 1412 cm^−1^ represents NH_4_^+^ bending in the IR spectrum.^34^ Du *et al.* reported that the IR peak at 1416 cm^−1^ was related to the bending vibration of the N–H bond.^30^Table 1 shows that the nine vibration modes from 1418 to 1673 cm^−1^ all represent NH_4_^+^ bending.

In the higher-frequency range above 2800 cm^−1^, the IR and Raman spectra exhibit several distinct characteristic peaks corresponding to N–H stretching vibrations. Waal *et al.* assigned vibrational peaks at 2839, 2926, and 3019 cm^−1^ to symmetric stretching, and peaks at 3122 and 3207 cm^−1^ to triply degenerate asymmetric stretching.^35^ Park *et al.* assigned Raman peaks at 2920 and 3050 cm^−1^ to NH_4_^+^ stretching.^36^ Onodera *et al.* observed IR peaks at 2800, 2980, and 3190 cm^−1^,^34^ while Du *et al.* assigned IR peaks at 2790, 2950 and 3200 cm^−1^ to NH_4_^+^ stretching.^30^ In this work, we identified that the modes at 2874, 2875, 2876, 3285, 3285, and 3289 cm^−1^ correspond to asymmetric stretching, while those at 2910, 2911, 2912, 2956, 3251, and 3253 cm^−1^ correspond to symmetric stretching. Although there are some high-intensity IR peaks in this range corresponding to N–H stretching modes, the PPRA effect does not directly facilitate V–O breaking. Two examples of normal modes at 1420 and 2876 cm^−1^ are shown in Fig. 4.

## Conclusions

Based on density functional theory simulations of the VDOS of NH_4_VO_3_, we analysed the dynamic processes of the normal modes. The results show that the IR-active modes and Raman-active modes are fully complementary. Each vibrational normal mode is either IR-active or Raman-active.

In particular, we confirmed that the normal modes from 711 to 994 cm^−1^ represent the V–O stretching vibrations. The highest-intensity peak in the IR spectrum is at 711 cm^−1^, indicating that the PPRA effect of IR radiation at this frequency is very strong. Although there are still some high-intensity peaks in the region above 2800 cm^−1^, they do not stimulate the PPRA effect of V-related vibrations.

V is typically obtained from V-bearing titanomagnetite and ilmenite ore^37^ through metallurgical processing, where it is produced as a by-product. The V in the ore is usually in the form of powdered V_2_O_3_, which is then dissolved in water or an acidic or alkaline solution to form V-containing ion clusters.^38–40^ The two main chemical methods currently used for the industrial extraction of V are the sodium roasting–water leaching process^9–13^ and the calcium roasting–acid leaching process.^14–18^ Based on our mode analysis, we propose the use of a high-power terahertz laser radiation at 711 cm^−1^ to assist in breaking the V–O bonds and separating V from NH_4_VO_3_. With the continuous progress of terahertz laser technology, this PPRA method could offer new application prospects. By utilising this PPRA physical method, it may be possible to achieve an environmentally friendly and efficient extraction of V from ores.

## Conflicts of interest

There are no conflicts to declare.

## Supplementary Material

RA-013-D3RA02053C-s001

## References

[cit1] Seron A., Menad N., Galle-Cavalloni P., Bru K. (2020). J. Sustain. Metall..

[cit2] Nazarov Yu. P., Vedyakov I. I., Odesskii P. D. (2007). Steel Transl..

[cit3] Zhen C., Mao X. P., Bao S. Q., Zhao G., Xu Y. W. (2019). Metals.

[cit4] Li Q. Z., Chen E. Y., Bice D. R., Dunand D. C. (2008). Metall. Mater. Trans. A.

[cit5] Agapovichev A., Sotov A., Kokareva V., Smelov V. (2018). MATEC Web Conf..

[cit6] Maedaa T., Shiraib Y. (2013). Mater. Sci. Forum.

[cit7] Yu V. K., Chen D. (2014). J. Power Sources.

[cit8] Sovacool B. K., Ali S. H., Bazilian M., Radley B., Nemery B., Okatz J., Mulvaney D. (2020). Science.

[cit9] Li M., Xiao L., Liu J. J., Shi Z. X., Fu Z. B., Peng Y., Long P. Z., Yang Y. J. (2016). Mater. Sci. Forum.

[cit10] Hu P. C., Zhang Y. M., Liu H., Liu T., Li S., Zhang R. B., Guo Z. J. (2023). Sep. Purif. Technol..

[cit11] Li H. Y., Fang H. X., Wang K., Zhou W., Yang Z., Yan X. M., Ge W. S., Li Q. W., Xie B. (2015). Hydrometallurgy.

[cit12] Zhang S. H., Li G. H., Xiao R. D., Luo J., Yi L. Y., Rao M. J. (2021). J. Mater. Res. Technol..

[cit13] Chen T. J., Zhang Y. M., Song S. X. (2010). Asia-Pac. J. Chem. Eng..

[cit14] Zhao Y. L., Chen L. C., Yi H., Zhang Y. M., Song S. X., Bao S. X. (2018). Minerals.

[cit15] Li Y., Peng Z. H., Wang Z. X., Zhu Y. Z., Xie K. Q. (2023). Minerals.

[cit16] Wen J., Jiang T., Liu Y. J., Xue X. X. (2019). Min. Proccess. Extr. Metall. Rev..

[cit17] Peng H., Li B., Shi W. B., Liu Z. H. (2022). Minerals.

[cit18] Zhang Y., Zhang T. A., Dreisinger D., Lv C. X., Lv G., Zhang W. G. (2019). J. Hazard. Mater..

[cit19] He D., Feng O., Zhang G., Luo W., Ou L. (2008). Miner. Metall. Proc..

[cit20] Ding M. T. (2021). IOP Conf. Ser.: Earth Environ. Sci..

[cit21] Lukesh J. S. (1950). Acta Cryst..

[cit22] Ekaterina S., Tamara K. (2016). Key Eng. Mater..

[cit23] Bencivenni L., Gingerich K. A. (1983). J. Mol. Struct..

[cit24] Twu J., Shih C. F., Guo T. H., Chen K. H. (1997). J. Mater. Chem..

[cit25] Zhang Y., Meisel M., Martin A., Lücke B., Witke K., Brzezinka K. W. (1997). Chem. Mater..

[cit26] Adams D. M., Haines J., Leonard S. (1991). J. Phys.: Condens. Matter.

[cit27] Waal D. D., Heyns A. M., Range K. J. (1990). Mat. Res. Bull..

[cit28] Zhang J., Feng Z., Li M., Chen J., Xu Q., Lian Y. X., Li C. (2007). Appl. Spectrosc..

[cit29] Ning W., Tang Z., Han Z., Ding S., Xu C., Zhang P. (2018). J. Ceram. Sci. Technol..

[cit30] Du G. C., Sun Z. H., Xian Y., Jing H., Chen H. J., Yin D. F. (2016). J. Cryst. Growth.

[cit31] Clark S. J., Segall M. D., Pickard C. J., Hasnip P. J., Probert M. I. J., Refson K., Payne M. C. (2005). Z. Kristallogr..

[cit32] Ernzerhof M., Scuseria G. E. (1999). J. Chem. Phys..

[cit33] Hawthorne F. C., Calvo C. (1977). J. Solid State Chem..

[cit34] Onodera S., Ikegami Y. (1980). Inorg. Chem..

[cit35] Waal D. D., Heyns A. M. (1990). Spectrochim. Acta.

[cit36] Park Y. S., Shurvell H. F. (1989). J. Raman Spectrosc..

[cit37] Moskalyk R. R., Alfantazi A. M. (2003). Miner. Eng..

[cit38] Gilligan R., Nikoloski A. N. (2020). Miner. Eng..

[cit39] Wen J., Jiang T., Yu T. X., Chen B. J., Li L. (2022). J. Clean. Prod..

[cit40] Liu S. Y., He X. B., Wang Y. D., Wang L. J. (2021). J. Clean. Prod..

